# A secreted protein of 15 kDa plays an important role in *Phytophthora palmivora* development and pathogenicity

**DOI:** 10.1038/s41598-020-59007-1

**Published:** 2020-02-11

**Authors:** Sittiporn Pettongkhao, Natasha Navet, Sebastian Schornack, Miaoying Tian, Nunta Churngchow

**Affiliations:** 1 Department of Biochemistry, Faculty of Science, Prince of Songkla University, Hat-Yai, Songkhla 90112 Thailand; 2 Department of Plant and Environmental Protection Sciences, University of Hawaii at Manoa, Honolulu, HI 96822 USA; 3 East-West Center, Honolulu, Hawaii USA; 4 Sainsbury Laboratory Cambridge University (SLCU), Cambridge, UK

**Keywords:** Microbiology, Plant sciences

## Abstract

*Phytophthora palmivora* is a destructive oomycete plant pathogen with a wide host range. So far, little is known about the factors governing its infection structure development and pathogenicity. From the culture filtrate of a *P. palmivora* strain isolated from papaya, we identified a secreted glycoprotein of 15 kDa, designated as Ppal15kDa, using liquid chromatography tandem mass spectrometry. Two gene variants, *Ppal15kDaA* and *Ppal15kDaB* were amplified from a *P. palmivora* papaya isolate. Transient expression of both variants in *Nicotiana benthamiana* by agroinfiltration enhanced *P. palmivora* infection. Six *Ppal15kDa* mutants with diverse mutations were generated via CRISPR/Cas9-mediated gene editing. All mutants were compromised in infectivity on *N. benthamiana* and papaya. Two mutants with all *Ppal15kDa* copies mutated almost completely lost pathogenicity. The pathogenicity of the other four containing at least one wild-type copy of *Ppal15kDa* was compromised at varying levels. The mutants were also affected in development as they produced smaller sporangia, shorter germ tubes, and fewer appressoria. The affected levels in development corresponded to the levels of reduction in pathogenicity, suggesting that Ppal15kDa plays an important role in normal development of *P. palmivora* infection structures. Consistent with its role in infection structure development and pathogenicity, *Ppal15kDa* was found to be highly induced during appressorium formation. In addition, Ppal15kDa homologs are broadly present in *Phytophthora* spp., but none were characterized. Altogether, this study identified a novel component involved in development and pathogenicity of *P. palmivora* and possibly other *Phytophthora* spp. known to contain a Ppal15kDa homolog.

## Introduction

Oomycetes are fungal-like microorganisms belonging to the kingdom Straminipila^1,2^. Their characteristics are distinct from true fungi since oomycetes are diploid or polyploid whereas fungi are haploid for most of the life cycles^2–4^. In addition, cell walls of oomycetes mainly consist of 1,3-beta-glucan, while the major cell wall constituent of fungi is chitin^5^. Oomycetes include many destructive plant pathogens, among which are over 100 species in the genus *Phytophthora* that severely threaten agricultural production and natural ecosystems^5,6^.

*Phytophthora palmivora* is a hemibiotrophic oomycete pathogen that infects more than 200 plant species in the tropics and subtropics^5^. Examples of economically important hosts include papaya, cacao, pineapple, durian, rubber tree, citrus, and oil palm. It also infects model plant species, such as *Nicotiana benthamiana* and *Medicago truncatula*^7–9^. Similar to other *Phytophthora* spp., plant infection by *P. palmivora* starts with motile zoospores, which encyst after contacting plant surfaces, followed by formation of germ tubes and then appressoria to penetrate the plant surface^7,10,11^. During infection, *P. palmivora* initially grows as a biotroph by forming haustoria inside the host cells to obtain nutrients, and then switches to necrotrophy in the later stages of infection^12,13^.

Elicitors have been shown to play a significant role in plant-pathogen interactions. During pathogen infection, plants are able to recognize pathogen-associated molecular patterns (PAMPs) or microbe-associated molecular patterns (MAMPs) to activate defense responses called PAMP-triggered immunity (PTI)^14^. PAMPs or MAMPs often derive from conserved components essential for pathogen survival and include a variety of proteins and other molecules^15^. Due to their defense-eliciting activities, they are also known as elicitors^15^. Many proteinaceous elicitors produced by *Phytophthora* spp. have been identified and characterized from culture filtrate. These elicitors are secreted proteins and some of them are also glycoproteins. Well-characterized examples include *P. infestans* elicitin INF1^16^, *P. parasitica* (current name: *P. nicotianae)* 34 kDa glycoprotein elicitor (CBEL)^17,18^, two glycoproteins of 32 kDa and 42 kDa from *P. megasperma*^19,20^, an oligopeptide of 13 amino acids (Pep-13) within the cell wall glycoprotein (GP42) of *P*. sojae^21^, and *P. sojae* glycoside hydrolase family 12 (GH12) protein XEG1^22^. In *P. palmivora*, the elicitin palmivorein and a 75 kDa protein were identified as elicitor proteins^23,24^. In addition, beta-glucan, high-molecular-weight glycoprotein, broad-molecular-weight glycoprotein and a 42-kDa elicitor were isolated from culture filtrate of *P. palmivora*^25^. Treatment of these elicitor proteins induces a range of plant defense responses on non-host and/or host plants, such as hypersensitive response (HR) which is characterized by rapid cell death, phytoalexin accumulation and defense-related gene expression^16–24^. As plants pretreated with elicitor proteins were shown to exhibit high levels of resistance^17,26^, identification of elicitors may promote the development of novel disease control methods. In addition, since some elicitor proteins, such as XEG1, also play an important role in virulence^22^, identifying proteins from cultrate filtrate of *Phytophthora* spp. may be able to reveal novel pathogenicity factors.

The application of CRISPR/Cas9-mediated gene editing technology has revolutionized oomycete functional genomics, which used to be very challenging due to their genetic features, such as being diploid or polyploid and heterothallic^27,28^. Since Fang and Tyler^29^ adapted this technology to oomycete genome editing, it has been successfully used to edit the genomes of three *Phytophthora* spp., including *P. sojae*, *P. capsici* and *P. palmivora*^28–30^. *Phytophthora palmivora*’s functional genomic studies particularly benefit from this technology as genome analyses of a cacao isolate suggested that *P. palmivora* is tetraploid^31^. Gumtow *et al*.^28^ used a simple and efficient *Agrobacterium*-mediated transformation method to express Cas9 and single guide RNA to achieve effective gene editing in *P. palmivora*. With this system, *P. palmivora* extracellular cystatin-like protease inhibitor *PpalEPIC8* mutants were successfully generated, which allowed the genetic identification of its role in pathogen virulence^28^. This system is expected to accelerate functional identification of many other *P. palmivora* effector proteins.

In this study, in search for potential elicitors from culture filtrate of *P. palmivora*, we identified a secreted glycoprotein of 15 kDa, designated as Ppal15kDa. We found that it plays an important role in *P. palmivora* development and pathogenicity. Transient expression of Ppal15kDa in *N. benthamiana* enhanced *P. palmivora* infection. *Ppal15kDa* mutants generated via CRISPR/Cas9 were compromised in infectivity on both *N. benthamiana* and papaya, which corresponded to their reduced sporangium sizes, impaired germ tube elongation and appressorium formation. *Ppal15kDa* was found to be highly expressed in appressorium-forming cysts, which is consistent with its role in infection structure development and pathogenicity.

## Results

### Identification of a 15 kDa glycoprotein from culture filtrate of *P. palmivora*

**Figure 1:**
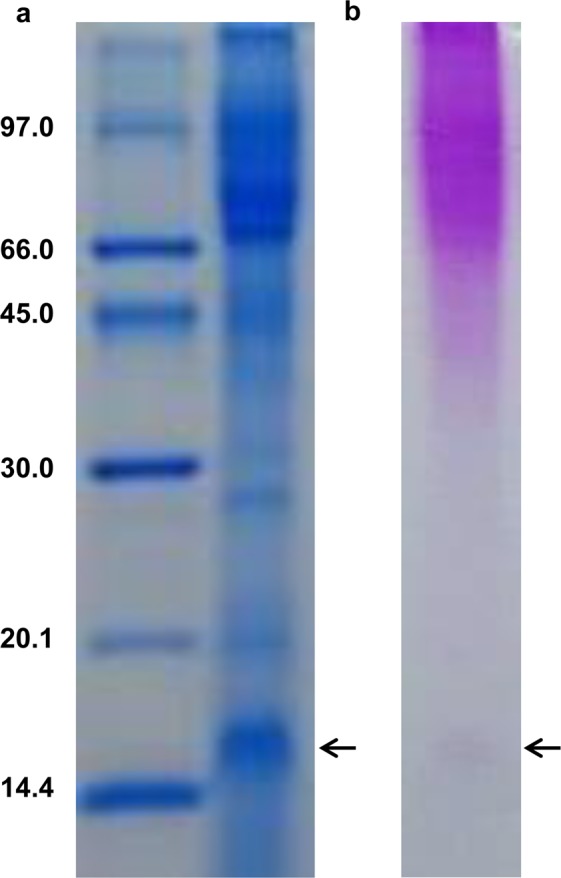
Detection of the 15 kDa glycoprotein from culture filtrate of *P. palmivora* (Full-length gels). The crude extract was separated by 15% SDS-PAGE and stained with InstantBlue Protein Staining (**a**) and periodic acid-Schiff reagent (**b**). The arrows indicate the 15 kDa protein.

To identify potential elicitors, culture filtrate of *P. palmivora* grown in Henniger medium^32^ was collected, dialyzed and lyophilized, and then separated on 15% SDS-PAGE. Two replicative gels were stained with InstantBlue Coomassie Protein Stain (Expedeon) and periodic acid-Schiff reagent for visualization of total proteins and glycoproteins, respectively. A strong protein band of about 15kDa appeared on the gel stained with InstantBlue Coomassie Protein Stain (Fig. 1a). A band of similar size also appeared on the gel stained with periodic acid-Schiff reagent (Fig. 1b). These suggest that there is a 15kDa glycoprotein abundantly present in the culture filtrate of *P. palmivora*.

**Figure 2:**
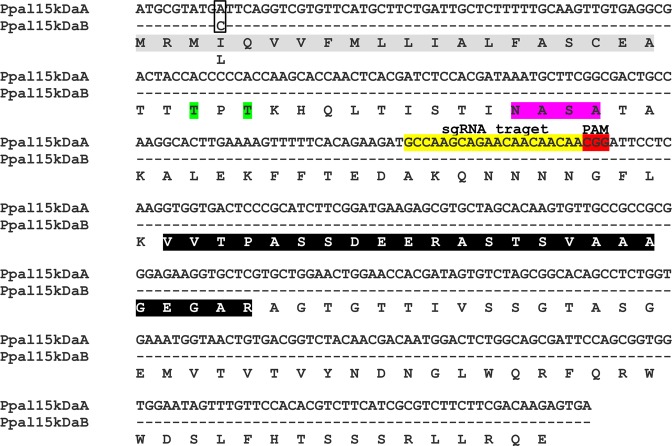
The nucleotide and amino acid sequences of *Ppa15kDaA* and *Ppal15kDaB* with the sgRNA target sequence used for CRISPR/Cas9-mediated gene editing. The dashed lines represent the same nucleotides in *Ppa15kDaA and Ppal15kDaB*. The single nucleotide polymorphism (SNP) leading to the substitution of isoleucine (I) and leucine (L) is shown in the box. The sgRNA target sequence together with its downstream protospacer adjacent motif (PAM) CGG is shown. The signal peptide sequence is shown in gray shade. The matching peptide sequences identified by LC-MS/MS are shown in black shade. The potential N-glycosylation (35 NASA) and O-glycosylation (Thr23 and Thr25) sites are highlighted in pink and green, respectively.

To identify this protein, the 15kDa band on the gel stained with InstantBlue Coomassie Protein Stain was cut and subjected to LC-MS/MS analyses using the *P. palmivora* transcriptome^13^ as the database. Multiple proteins (peptide score) were matched, including PLTG_02159.1 (68), PLTG_04166.1 (30), PLTG_11937.1 (29), PLTG_07335.1 (28), PLTG_10394.1 (24), PLTG_10965.2 (23), PLTG_11247.2 (21) and PLTG_04306.1 (21). However, the threshold peptide score of a protein with 95% identity should be higher than 31 (identity threshold). Only PLTG_02159.1 showed peptide score (68) higher than identity threshold (>31) and its molecular weight was 14.83 kDa. Two peptides VVTPASSDEER and ASTSVAAAGEGAR matched PLTG_02159.1^13^ with 100% identity (Fig. 2). Consequently, the PLTG_02159.1 protein was designated as Ppal15kDa.

We amplified and cloned *Ppal15kDa* from *P. palmivora* papaya isolate P1 using primers designed based on the nucleotide sequence of PLTG_02159.1^13^. We found two gene variants of *Ppal15kDa*, A and B forms (Fig. 2). A single nucleotide variation led to an amino acid change at the 4th amino acid from the N-terminus with isoleucine in A and leucine in B form (Fig. 2). The translated amino acid of the *Ppal15kDa* had 136 residues with a putative signal peptide of 20 amino acids predicted using Signal P 5.0^33^, which identified an Sec/SPI type secretory signal peptide with a likelihood of 0.9994 and the cleavage cite between amino acid position 20 and 21 with a probability of 0.8875 (Fig. 2). There were a potential N-glycosylation (Asn-X-Ser/Thr) site (35 NASA) and two potential O-glycosylation sites (Thr23 and Thr25) (Fig. 2).

The homologs of Ppal15kDa appeared to be broadly present in plant pathogenic *Phytophthora* spp. (Fig. S1). Using BLASTP and TBLASTN against NCBI non-redundant database, and the protein, transcript, EST and PopSet databases in FungiDB (https://fungidb.org/fungidb/), homologous sequences of Ppal15kDa were found in *P. megakarya*, *P. cactorum*, *P. parasitica* (current name: *P. nicotianae*), *P. sojae*, *P. cinnamomi* and *P. capsici* with identify higher than 50% and E-value below 1e-22 (Fig. S1). All these sequences were annotated as hypothetical proteins. The phylogenetic dendrogram revealed that Ppal15kDa was most closely related to the hypothetical protein OWZ12091.1 from *P. megakarya* (Fig. S2). No functional domain indicative of Ppal15kDa’s biochemical function was identified using NCBI Conserved Domain Search and InterProScan by searching multiple databases that make up the InterPro consortium^34^.

### Expression of *Ppal15kDa* in *N. benthamiana* enhances *P. palmivora* infection

**Figure 3:**
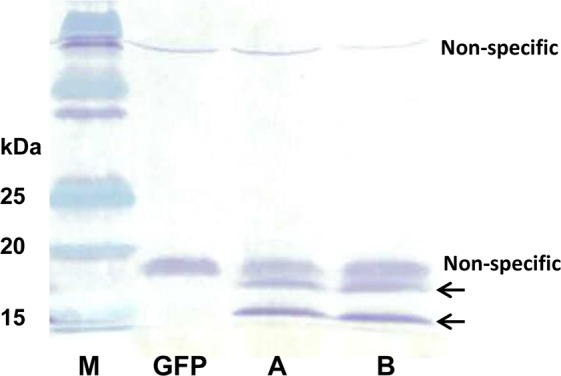
Agrobacterium-mediated transient expression of *Ppal15kD*a in *N. benthamiana*. Total proteins were extracted from infiltrated *N. benthamiana* leaves and subjected to SDS-PAGE followed by Western blot with HRP conjugated anti-His monoclonal antibody. Lane M represents the protein standard and lane GFP represents *N. benthamiana* leaves infiltrated with *A. tumefaciens* GV3101 carrying pJL-TRBO-G. Lane A and B represent *N. benthamiana* leaves infiltrated with *A. tumefaciens* GV3101 carrying pJL-TRBO-*Ppal15kDaA* and pJL-TRBO-*Ppal15kDaB*, respectively. The arrows indicate two forms of Ppal15kDa. The full-length gel is presented in Supplementary Fig. 3 (Fig. S3).

To assess the roles of Ppal15kDa in pathogen virulence, we expressed Ppal15kDa in *N. benthamiana* via Agrobacterium-mediated transient expression. The *Ppal15kDaA* and *Ppal15kDaB* coding sequences fused to the hexahistidine (His)-tag at the C-terminus were cloned into pJL-TRBO plasmid^35^ and transiently expressed in *N. benthamiana* leaves by agroinfiltration. The expression of Ppal15kDaA and Ppal15kDaB proteins were analyzed in total proteins extracted from agroinfiltrated *N. benthamiana* leaves by Western blot with horse radish peroxidase (HRP) conjugated anti-His monoclonal antibody. Two bands of approximately 15 kDa and 17 kDa were detected in leaves infiltrated with *Agrobacteria* carrying pJL-TRBO-Ppal15kDaA or pJL-TRBO-Ppal15kDaB, but not in leaves infiltrated with Agrobacteria carrying pJL-TRBO-GFP for expression of GFP (Fig. 3), demonstrating the successful expression of 15 kDa. The two bands of 15 kDa and 17 kDa may represent different modifications of the proteins.

**Figure 4:**
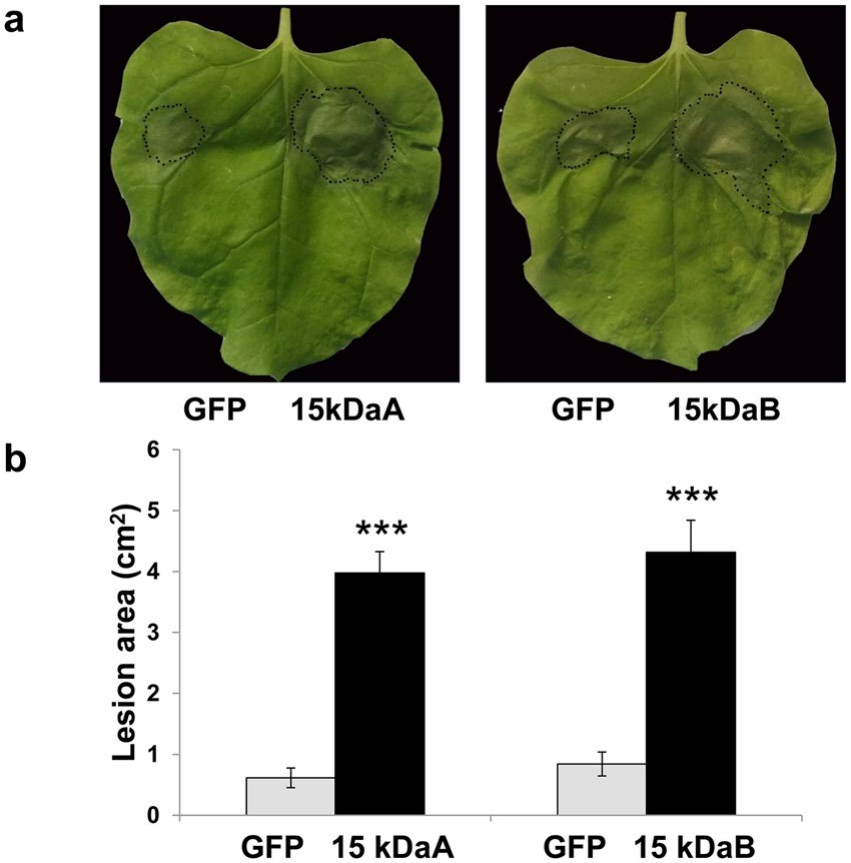
Expression of Ppal15kDa in *N. benthamiana* leaves enhanced *P. palmivora* infection. (**a**) Infection symptoms of *P. palmivora* on a representative *N. benthamiana* leaf with one half transiently expressing Ppal15kDaA or Ppal15kDaB and the other half expressing GFP. The photograph was taken at 4 days post inoculation (dpi). (**b**) The average lesion areas caused by *P. palmivora* at 4 dpi on *N. benthamiana* leaves treated as in (**a**). The histograms correspond to the mean ± standard errors (SE) of lesion areas calculated from independent leaves (n = 28). Three asterisks (***) indicate statistically significant differences (*P*-value < 0.001) in the lesion areas on leaf halves expressing Ppal15kDaA or Ppal15kDaB compared to the other halves expressing GFP determined by paired t-test.

*N. benthamiana* leaf halves expressing the Ppal15kDa or GFP were inoculated with zoospore suspensions and lesions were measured at 4 days post inoculation. Lesions on *N. benthamiana* half leaves expressing Ppal15kDaA or Ppal15kDaB were much larger than the ones expressing GFP (Fig. 4a,b). Average lesion sizes of *N. benthamiana* leaf halves expressing Ppal15kDaA and Ppal15kDaB were 3.9 and 4.5 cm^2^, respectively, which were about four fold higher than the control (Fig. 4b). This result indicated that Ppal15kDa contributed to *P. palmivora* virulence during infection.

### Generation of *Ppal15kDa* mutants by CRISPR/Cas9-mediated gene editing

To further confirm the role of Ppal15kDa in *P. palmivora* virulence, we generated Ppal15kDa mutants using CRISPR/Cas9-mediated gene editing. A 20-nt sgRNA target sequence targeting the upstream of Ppal15KDa central region was selected (Fig. 2), which together with the HH ribozyme sequence^27^ were cloned into pCB301TOR-CRISPR. The resulted plasmid pCB301TOR-CRISPR-*Ppal15kDa* was used to transform *P. palmivora* via *A. tumefaciens*-mediated transformation. We isolated single zoospore-derived transformants from 20 initial transformants and sequenced *Ppal15kDa* to identify the mutations. In *Ppal15kDa* sequencing chromatograms, we observed mixed peaks immediately after the Cas9 cleavage site in six single zoospore lines T1-2, T3-1, T9-4, T11-10, T13-2 and T17-5, but not in WT (Fig. S4), suggesting that mutations occurred in these lines. These lines originated from six initial transformants T1, T3, T9, T11, T13 and T17 and therefore represent independent transformation events. The mixed sequence chromatograms suggested that not all copies of *Ppal15kDa* in the genome were mutated and/or different mutations occurred at different copies (Fig. S4).

**Figure 5:**
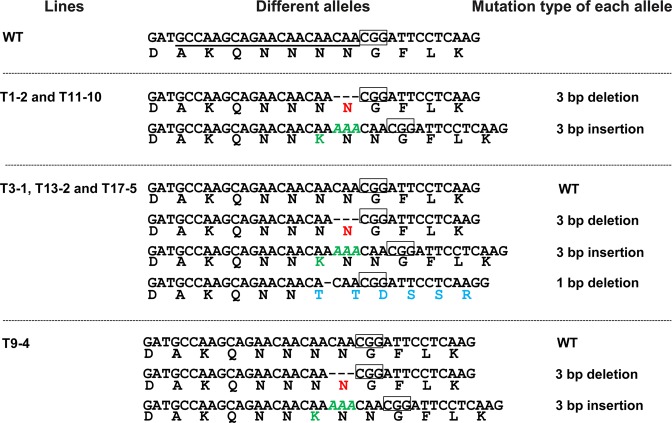
The *Ppal15kDa* nucleotide mutations and the resulted amino acid changes in *P. palmivora* mutants generated via CRISPR/Cas9 gene editing. The sgRNA target sequence in WT is underlined and PAM motif is shown in the box. The inserted nucleotides are shown in green italic and the deleted nucleotides are indicated with dashed lines. The deleted, inserted and changed amino acids are shown in red, green and blue, respectively.

To determine the types of mutations in the above transformants, we cloned the PCR products of *Ppal15kDa* from these transformants and performed Sanger sequencing. The number of sequenced clones for each transformant varied from 12–24. Two types of mutations were observed in T1-2 and T11-10 transformants, including a 3 bp insertion leading to the addition of lysine (K) and a 3 bp deletion leading to the loss of asparagine (N) (Fig. 5). T3-1, T13-2 and T17-5 had the wild-type (WT) copy and three types of mutations, including the two types that occurred in T1-2 and T11-10 and 1 bp deletion resulting in frame shift (Fig. 5). T9-4 had the WT copy and the two types of mutations in T1-2 and T11-10 (Fig. 5).

### *Ppal15kDa* mutants are compromised in pathogenicity

**Figure 6:**
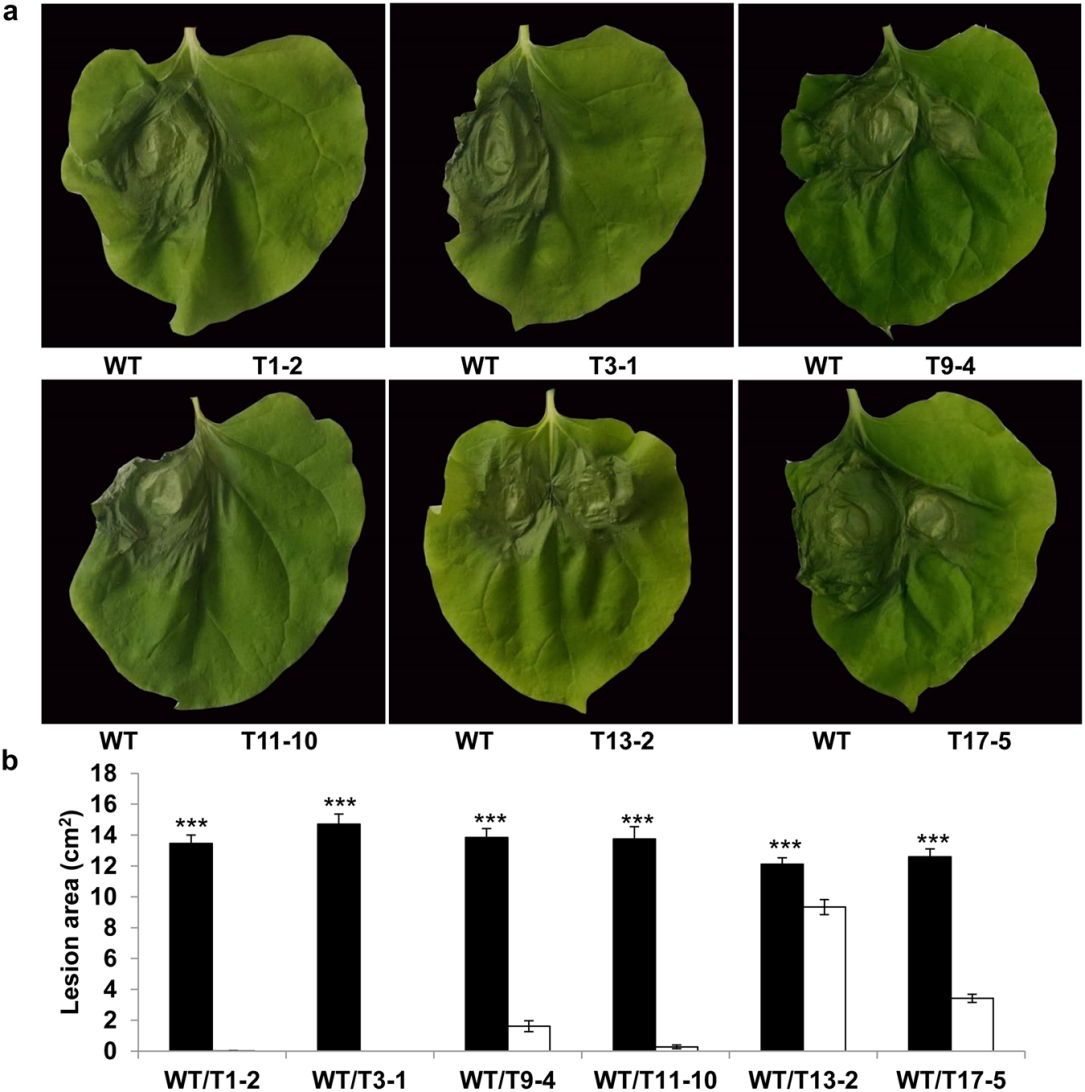
Infection assays of *N. benthamiana* leaves with *P. palmivora* wild-type (WT) strain and *Ppal15kDa* mutants. (**a**) Symptoms of representative leaves infected with WT and mutant strains side-by-side at 4 days post inoculation (dpi). (**b**) The average lesion areas at 4 dpi on *N. benthamiana* leaves inoculated as in (**a**). The histograms correspond to the mean ± standard errors (SE) of lesion areas calculated from independent leaves (n = 20). Three asterisks (***) indicate statistically significant differences (*P*-value < 0.001) in the lesion areas caused by WT compared to *Ppal15kDa* mutants determined by paired t-test.

To investigate the effect of *Ppal15kDa* mutations on *P. palmivora* pathogenicity, six Ppal15kDa mutants were inoculated on *N. benthamiana* leaves and papaya fruit side-by-side with WT. Inoculation of *N. benthamiana* leaf halves with WT consistently produced large lesions. In contrast, the *N. benthamiana* leaf halves inoculated with different mutants produced lesions of varying sizes that were smaller than WT (Fig. 6a). T1-2, T3-1 and T11-10 showed either no infection or very small lesions (Fig. 6a). In this study, the range of lesion areas cause by WT was 12.6 to 14.7 cm^2^. The average lesion areas caused by T1-2, T3-1 and T11-10 were 0.1, 0 and 0.3 cm2, respectively (Fig. 6b). T9-4 and T17-5 caused larger lesions on *N. benthamiana* leaves than T1-2, T3-1 and T11-10, but significantly smaller than WT. The average lesion areas of T9-4 and T17-5 were 1.6 and 3.4 cm2, respectively (Fig. 6b). The lesions caused by T13-2 were only slightly smaller than WT (Fig. 6a,b).

**Figure 7:**
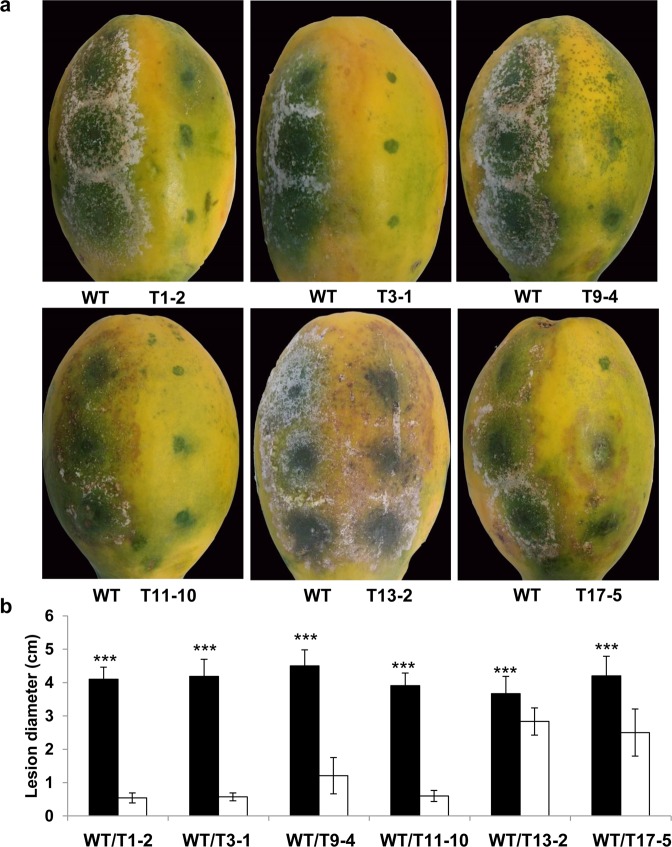
Infection assays of papaya fruits with *P. palmivora* wild-type (WT) strain and *Ppal15kDa* mutants. (**a**) Symptoms of representative papaya fruits infected with WT and mutant strains side-by-side at 4 dpi. (**b**) The average lesion diameters at 4 dpi on papaya fruits inoculated as in (**a**). The histograms correspond to the mean ± standard errors (SE) of lesion diameters calculated from independent fruits (n = 13). Three asterisks (***) indicate statistically significant differences (*P*-value < 0.001) between WT and the indicated *Ppal15kDa* mutant determined by paired t-test.

Similar results were obtained from infection assays of papaya fruit. In this study, the range of lesion diameters caused by WT was 3.85 to 4.42 cm. The lesions caused by mutants, T1-2, T3-1, T9-4, T11-10 and T17-5 were significantly smaller than WT (Fig. 7a), with the average lesion diameters as 0.54, 0.55, 1.19, 0.8, and 2.31, respectively (Fig. 7b). T13-2 produced lesions slightly smaller than WT, with the average lesion diameter as 2.94 (Fig. 7b).

To exclude the possibility that *P. palmivora* transformation with the construct for gene editing might have contributed to the reduced disease in gene-edited mutants, we inoculated *N. benthamiana* leaf halves with WT and four representative lines that went through transformation but did not have mutations in *Ppal15kDa*. We did not observe the difference of infection areas caused by WT and these transgenic lines without successful gene editing (Fig. S5), suggesting that the reduced lesions in *Ppal15kDa* mutants were indeed attributed to the mutations in *Ppal15kDa*. In summary, although the infectivity of different mutants varied, they all exhibited reduced virulence compared with WT with some mutants’ virulence almost completely compromised. These results suggest that *Ppal15kDa* gene plays an important role in *P. palmivora* pathogenicity.

### *Ppal15kDa* mutants produce smaller sporangia and are compromised in germ tube elongation and appressorium formation

Because *Ppal15kDa* mutants are compromised in pathogenicity on both *N. benthamiana* leaves and papaya fruits, we investigated whether mutations of *Ppal15kDa* affect *P. palmivora* development. We did not observe mutations of Ppal15kDa cause changes in mycelium growth (Fig. S6), sporulation (All mutants could produce sporangia) and zoospore germination (Fig. S7). However, we observed significant difference in sporangium sizes, germ tube lengths and appressorium formation between WT and Ppal15kDa mutants. We used five mutants (T1-2, T3-1, T9-4, T13-2 and T17-5) for these assays. As T1-2 and T11-10 exhibited similar genotype and virulence, we only included one of them (T1-2).

**Figure 8:**
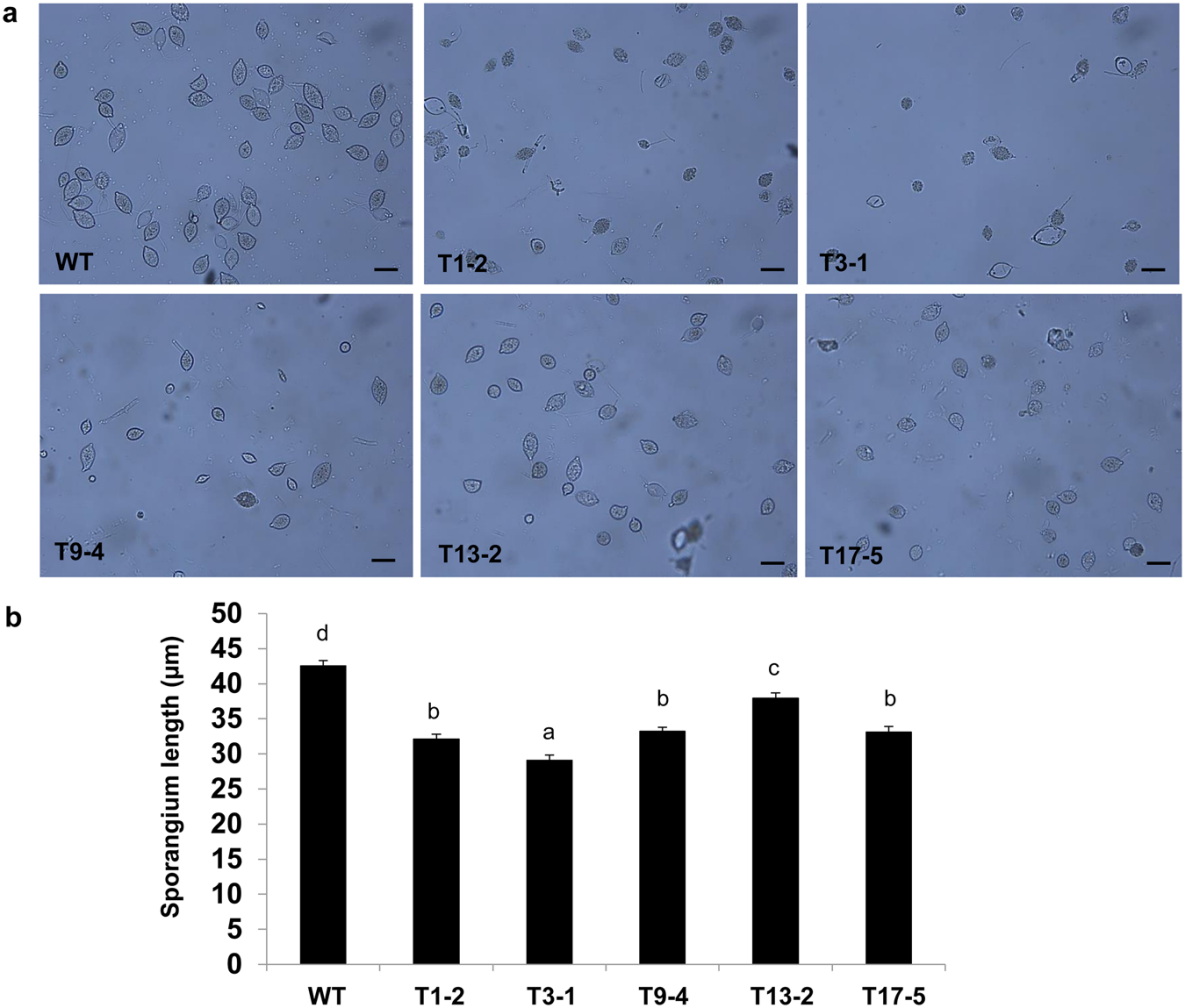
Measurements of the sporangium sizes of *P. palmivora* WT strain and *Ppal15kDa* mutants. (**a**) Representative micrographs of sporangia of WT and mutant strains (scale bars = 50 µm). (**b**) The average sporangium length of wild-type (WT) strain and *Ppal15kDa* mutants. The histograms correspond to the mean ± standard errors (SE) of sporangium length calculated from independent sporangium (n = 120). Different letters indicate significant differences, determined using Duncan’s multiple range test (*P*-value < 0.05).

The sporangium sizes of all tested mutants were found to be significantly smaller than WT (Fig. 8). The average sporangium lengths of T1-2, T3-1, T9-4, T13-2 and T17-5 were 32, 29, 33, 37, 33 µm, respectively, whereas the average sporangium length of WT was 42 µm (Fig. 8).

**Figure 9:**
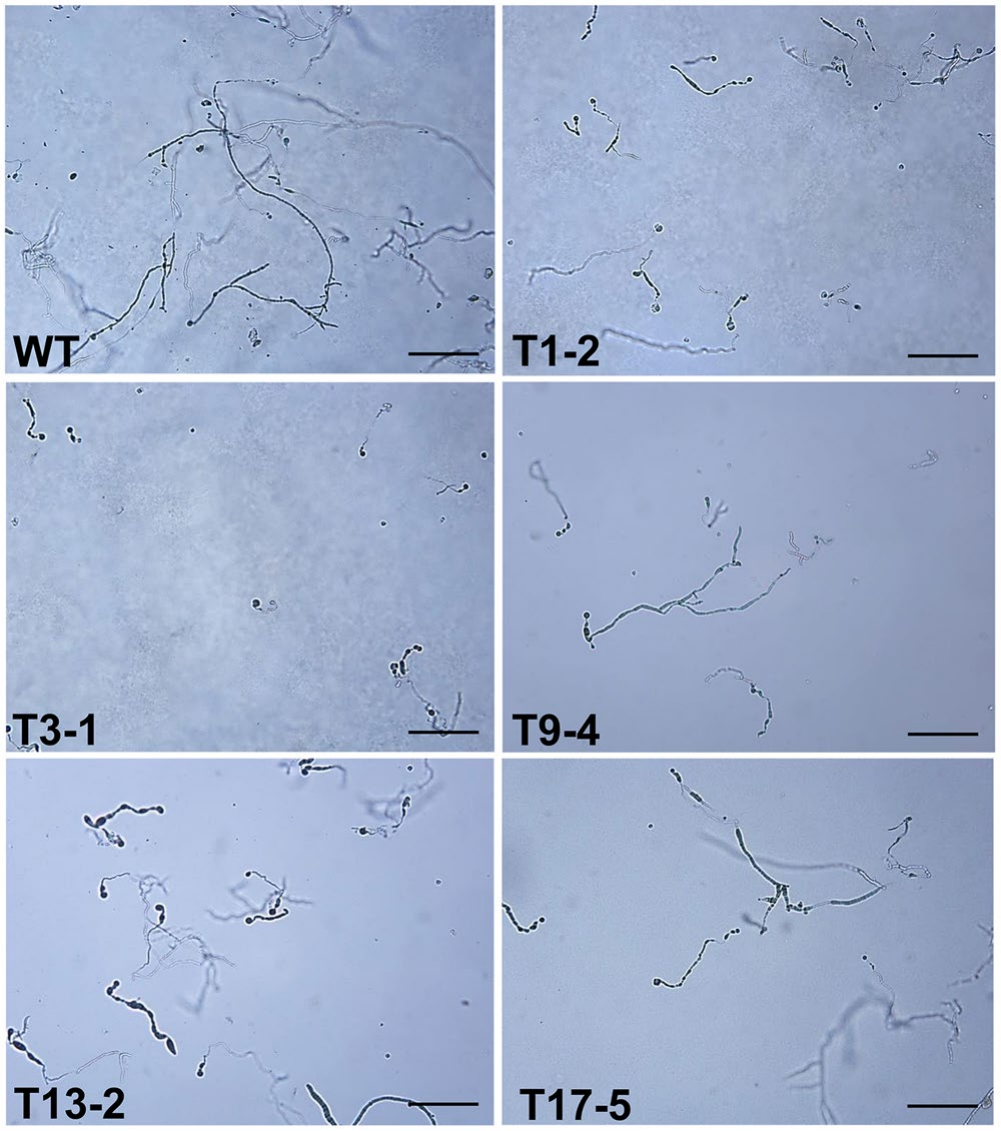
Micrographs of germ tubes of *P. palmivora* WT strain and *Ppal15kDa* mutants. Zoospores were cultured on Plich agar for 24 hours and photographed under light microscope (scale bars = 250 µm). Three experiments were performed.

We observed the zoospore germination and germ tube elongation under light microscope at 4 and 24 hours after incubating zoospores on Plich agar. At 4 hours, zoospore germination rate and germ tube lengths of WT and the mutants were not clearly different (Fig. S7). However, at 24 hours, the germ tubes of *Ppal15kDa* mutant T1-2, T3-1 and T9-4 appeared to be significantly shorter than WT, whereas the germ tube elongation of T13-2 and T17-5 was not clearly affected (Fig. 9).

**Figure 10:**
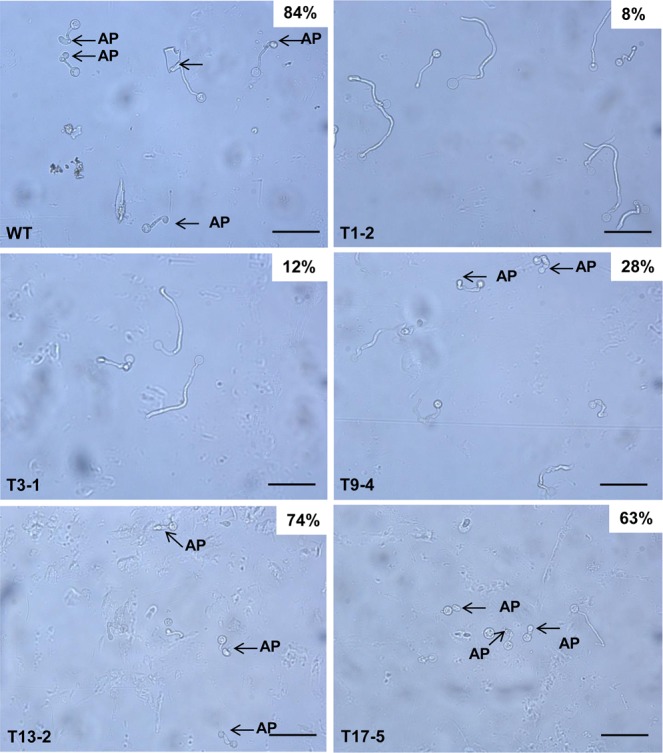
Assays of appressorium formation of *P. palmivora* WT and *Ppal15kDa* mutants. Zoospores were incubated between two plastic cover slips placed on Plich agar at room temperature for 4 hours. Appressoria (AP) were observed and counted under light microscope. The percentage of more than 200 germinated cysts that developed appressoria was shown at the upper right corner of each micrograph (scale bars = 50 µm). The experiment was repeated 2 times with similar results.

Appressoria were observed and counted under light microscope after 4 hours of induction by incubating zoospores between hydrophobic plastic cover slips, which were placed on Plich agar. The percentages of appressorium-forming cysts of T1-2, T3-1 and T9-4 were 8, 12 and 28%, which were much lower than WT with a percentage of 84% (Fig. 10). The percentages of appressorium-forming cysts of T13-2 and T17-5 were 74 and 63%, which were slightly lower than WT (Fig. 10).

Clearly, the extent to which germ tubes and appressoria in the mutants were affected was consistent with the level of reduced pathogenicity on plants. T1-2, T3-1 and T9-4 were dramatically compromised in pathogenicity on *N. benthamiana* and papaya (Figs. 6 and 7), and their germ tube elongation and appressorium formation were affected at a high level. Consistently, the pathogenicity of T13-2 and T17-5 was affected to a lesser extent, and correspondingly their germ tube elongation and appressorium formation were either not clearly affected or slightly affected. Altogether, the above data suggest that Ppal15kDa plays a significant role in sporangium formation and development of infection structures such as germ tubes and appressoria, and Ppal15kDa contributes to pathogenicity at least in part by functioning in normal development of *P. palmivora* infection structures.

### *Ppal15kDa* is highly induced during appressorium formation

**Figure 11:**
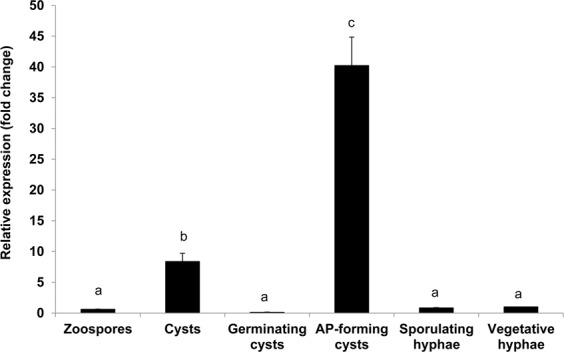
Expression of *Ppal15kDa* in various development stages of *P. palmivora* by RT-qPCR. cDNAs were synthesized from RNA extracted from zoospores, cysts, germinating cysts, appressorium-forming cysts, sporulating hyphae, and vegetative hyphae of *P. palmivora*. The expression was normalized using *P. palmivora β-tubulin* gene and relative to vegetative hyphae with the expression level in vegetative hyphae calculated as 1. The error bars represent standard deviations from three technical replicates. Different letters indicate significant differences, determined using Duncan’s multiple range test (*P*-value < 0.05).

We determined the expression of *Ppal15kDa* at various *P. palmivora* developmental stages, including zoospores, cysts, germinating cysts, appressorium-forming cysts, sporulating hyphae and vegetative hyphae, using quantitative reverse transcription PCR (RT-qPCR). The expression of *Ppal15kDa* in appressorium-forming cysts was the highest among all tested developmental stages (Fig. 11) and induced by about 40 fold compared to the expression in vegetative hyphae (Fig. 11). In addition, the expression of *Ppal15kDa* was also induced in cysts by about 8 fold. This result suggests that *Ppal15kDa* is expressed at early stage of infection, i. e. appressorium-forming cysts, a stage before and when penetration takes place.

## Discussion

Many proteins that play important roles in plant-oomycete interactions have been identified from culture filtrate of *Phytophthora* spp^16–25,36–38^. For example, *P. sojae* glycoside hydrolase family 12 (GH12) protein, XEG1, identified from culture filtrate, acts as an important virulence factor during infection but also serves as a PAMP to trigger defense responses including cell death^22^. In this study, we identified a secreted protein of 15 kDa, Ppal15 kDa, abundantly present in culture filtrate of *P. palmivora* using LC-MS/MS. By transient expression of Ppal15kDa in *N. benthamiana* and generation of its *P. palmivora* mutants using CRISPR/Cas9 gene editing, we found that Ppal15kDa plays a significant role in *P. palmivora* infection structure development and pathogenicity. Ppal15kDa is a previously uncharacterized protein. Besides the signal peptide, it does not have a functional domain indicative of its biochemical function and its homologs were not found in organisms other than *Phytophthora* spp. *Ppal15kDa* homologs found in *Phytophthora* spp. were all annotated as a hypothetical protein. As such, this study identified a novel secreted protein involved in development and pathogenicity of *P. palmivora* and possibly many other *Phytophthora* spp. as *Ppal15kDa* homologs appear to be broadly present in *Phytophthora* spp. In addition, as Ppal15kDa is an extracellular protein and its mutations almost completely crippled *P. palmivora* pathogenicity, it represents a potential ideal target for genetic and chemical control.

After landing and attaching to the host, *P. palmivora* zoospore germinates and produces a germ tube, which differentiates into an appressorium to penetrate the host surface^12,13^. Our results showed that Ppal15kDa mutants were impaired in germ tube elongation and appressorium formation, which corresponded to their compromised pathogenicity, suggesting that Ppal15kDa contributes to infection at early stage by participating in infection structure development. In support of this, *PLTG_02159* which is the Ppal15kDa-encoding gene, was found to be induced at 6 hours post infection, but not at later hours^13^. Many plant pathogenic fungi secrete an extracellular matrix (ECM) that is associated with germ tubes, spores and hyphae^39^. It has been reported that the extracellular matrix protein EMP1 plays an important role in appressorium formation and pathogenicity of the rice blast fungus, *Magnaporthe grisea*. An *EMP1* null mutant generated by targeted gene disruption showed reduced levels of appressorium formation and pathogenicity^40^. EMP1 contains an 18-amino-acid N-terminal secretion signal peptide and four putative N-glycosylation sites^40^. Similarly, Ppal15kDa consists of a 20-amino-acid N-terminal secretion signal peptide (Fig. 2), one potential N-glycosylation (Asn-X-Ser/Thr) site (35 NASA) and two potential O-glycosylation sites (Thr23 and Thr25) (Fig. 2). Northern blot analysis displayed that *EMP1* transcript levels were accumulated during appressorium formation but not during vegetative growth^40^. Like *EMP1*, *Pal15kDa* was induced during appressorium formation but not during vegetative growth (Fig. 11).

Two specific bands of 15 and 17 kDa were detected by Western blot in *N. benthamiana* leaves expressing His-tagged Ppal15kDa (Fig. 3). They likely represent two different glycosylated forms of Ppal15kDa as Ppal15kDa can be stained using periodic acid-Schiff reagent (Fig. 1), which is used for staining of glycoproteins, and has potential N-glycosylation and O-glycosylation sites (Fig. 2). Many glycoproteins expressed in plants exhibited two bands on SDS-PAGE. For example, McGarvey *et al*.^41^ expressed a glycoprotein (G-protein) that coats the outer surface of rabies virus in tomato using *A. tumefaciens*-mediated transformation, and Western blot detection revealed two distinct bands with apparent molecular weight of 60 and 62 kDa. After mixing the G-protein of higher molecular weight with extracts from a WT tomato plant, the smaller size was observed. Their results suggest that a specific enzymatic cleavage of sugar or amino acid residues may be responsible for double bands^41^. Zhang *et al*.^42^ expressed the cell wall glycoprotein (SP)_32_ fused with green fluorescence protein in the leaves and hairy roots of tobacco (*N. tabacum*). They observed two bands of 40 and 42 kDa. The 42 kDa band disappeared when the expressed product was subjected to β-elimination treatment known to remove sugars O-linked to Ser/Thr^42^. When N-glycosylated cholera toxin B subunit (gCTB) was transiently expressed in *N. benthamiana* leaves, Western blot analysis of the expressed protein without glycosidase treatment showed two distinct bands^43,44^. The band with the higher molecular weight corresponds to the glycosylated form of gCTB whereas another band represents the aglycosylated form^43^.

We do not have direct evidence suggesting that Ppal15kDaA and Ppal15kDaB are homoalleles or paralogs in *P. palmivora* genome, but indirect evidence suggests the former. The Ppal15kDa-encoding sequence was found in a single scaffold of the genome assembly of a tetraploid *P. palmivora* cacao isolate^31^ using BLAST search. In addition, in the Ppal15kDa mutants generated using CRISPR/Cas9 gene editing, the maximal number of different versions of Ppal15kDa observed in a single zoospore-derived transformant is four (Fig. 5).

We tested 6 independent single zoospore-derived Ppal15kDa mutants for their infectivity on *N. benthamiana* and papaya and analyzed their phenotypes at various developmental stages. Although overall these mutants were compromised in pathogenicity and affected in sporangium sizes, germ tube elongation and appressorium formation, the affected levels varied. In T1-2 and T11-10, all *Ppal15kDa* copies were mutated. Correspondingly, these two mutants almost completely lost pathogenicity and the development was considerably affected. In the remaining four mutants (T3-1, T9-4, T13-2, and T17-5), there was at least one wild-type (WT) *Ppal15kDa* copy present, as a result, their pathogenicity and development were less affected than T1-2/T11-10 except T3-1. T3-1, T13-2 and T17-5 seem to have the same pattern of gene editing (Fig. 5), however, their phenotypes varied significantly. There are several factors that might contribute to the difference. When we sequenced the *Ppal15kDa* gene in the mutants, the primers used did not differentiate Ppal15kDaA and Ppal15kDaB versions. Therefore, although we observed the same pattern of mutations and WT copy, as these mutations (or WT) could be in either Ppal15kDA or Ppal15kDaB, these mutants are very likely to be different regarding their expression patterns and levels, although Ppal15kDaA and Ppal15kDaB do not seem to be functionally divergent at the protein level as both were equally effective in enhancing *P. palmivora* infection (Fig. 4). In this study, we were not able to sequence 5′ UTR (Untranslated region) and the promoter region of *Ppal15kDaA* and *Ppal15kDaB* due to the constraint of the duration and amount of the funding. Future study should identify this region and clarify this matter. The difference contributed by these two versions may lie in their different transcription and/or translation efficiency. The *Ppal15kDa* copy with higher expression may not have been edited and this wild-type copy likely contributed to the pathogenicity of the mutants that were less affected. In most cases, two chromosomal copies (both alleles) of a gene are transcribed called bi-allelic expression. However, some genes display monoallelic expression which only one allele of a gene is expressed. For *P. palmivora*, it is tetraploid. Possibly, the allele which is highly expressed did not get edited. Other factors that contribute to the differences among T3-1, T13-2 and T17-5 could be different integration sites of the T-DNA expressing Cas9 and Ppal15kDa-targeting sgRNA, which affected the phenotypes due to disruption or interference of the neighboring genes. Integration of this construct may enhance or reduce expression of some genes which is important for pathogenicity of *P. palmivora*.

This study revealed the roles of *Ppal15kDa* including promoting infection, germ tube elongation, sporangium development and appressorium formation. However, the underlying mechanisms remain to be investigated. Identification of the interacting proteins of Ppal15kDa from both *P. palmivora* and the host plants would be a good start to reveal them in the future.

## Materials and Methods

### *P. palmivora* growth and zoospore preparation

*P*. *palmivora* P1 strain isolated from a naturally infected papaya plant^45^ was used throughout this study. It was routinely cultured on 10% unclarified V8 agar (10% V8 juice, 0.1% CaCO_3_, 1.5% agar) at 25 °C for 7 days. For preparing zoospore suspensions used in various assays, the 7-day-old culture in a 100 mm Petri dish was flooded with twelve milliliters of cold (4 °C) sterile distilled water. The plate was incubated at 4 °C for 15 min and then at room temperature for 15 min to release zoospores. The concentration of zoospores was measured using a haemocytometer under a light microscope.

### N. benthamiana growth

*N*. *benthamiana* plants were grown in a growth chamber at 25 °C, 60% humidity, under 12h-light and 12h-dark cycle. Six-week-old *N*. *benthamiana* plants were used for all agroinfiltration experiments and infection assays with wild-type (WT) and mutant *P. palmivora*.

### Identification of Ppal15kDa in *P. palmivora* culture filtrate

For preparation of *P. palmivora* culture filtrate, 7-day-old *P. palmivora* on 10% V8 agar was cut with a cork borer, and then cultured in Henniger liquid medium^32^ at 25 °C with shaking at 100 rpm for 14 days. The liquid culture was filtered through Whatman filters (No. 1), dialyzed with a dialysis membrane with a cutoff of 12 kDa, and then lyophilized. The culture filtrate proteins were separated on 15% SDS-PAGE and stained with InstantBlue Protein Stain kit (Expedeon, U.K.) following the manufacturer’s protocol. The protein band of around 15 kDa was cut and subsequently identified by liquid chromatography tandem mass spectrometry (LC-MS/MS) using a database generated from *P. palmivora* transcriptomic sequences^13^. In order to stain glycoproteins, periodic acid-Schiff staining^46^ was performed by soaking the gel in 7.5% (v/v) acetic acid for 1 hour, and then in 1% (w/v) periodic acid for 45 min at 4 °C in the dark, followed by washing in 7.5% acetic acid for 10 min (6 times), staining with the periodic acid-Schiff reagent for 1 hour at 4 °C in the dark and then washing with 0.5% (w/v) sodium metabisulfite.

### Bacterial strains and plasmids

*Agrobacterium tumefaciens* strains GV3101^47^ and EHA105 and *E. coli* strain DH5α (Invitrogen, CA, USA) were grown on Luria-Bertani (LB) agar or broth supplemented with appropriate antibiotics^48^ at 28 °C or 37 °C, respectively. The pGEM-T vector (Promega, Madison, WI, USA) was used to clone *Ppal15kDa*. pJL-TRBO, a *Tobacco Mosaic Virus* (TMV)-based binary vector that allows high levels of expression of foreign proteins in plants^35^, was used to transiently express *Ppal15kDa* in *N. benthamiana* leaves. pCB301TOR-CRISPR^28^ was used for CRISPR/Cas9-mediated gene editing of *Ppal15kDa* in *P. palmivora*.

### Bioinformatics analysis

Protein translation was performed using the translate tool (ExPASy; http://web.expasy.org/translate/). Prediction of signal peptide was conducted using SignalP version 5.0 (http://www.cbs.dtu.dk/services/SignalP/)^33^. Potential O- and N-glycosylation sites were predicted using DictyOGlyc version 1.1 (http://www.cbs.dtu.dk/services/DictyOGlyc/)^49^ and NetNGlyc version 1.0 (www.cbs.dtu.dk/services/NetNGlyc/)^50^, respectively. BLASTP and TBLASTN were performed using tools and databases in NCBI BLAST server (https://blast.ncbi.nlm.nih.gov/Blast.cgi)^51^ and FungiDB (https://fungidb.org/fungidb/)^52^. Conserved or functional domain search was performed using NCBI Conserved Domain search engine (https://www.ncbi.nlm.nih.gov/Structure/cdd/wrpsb.cgi)^53^ and InterProScan (http://www.ebi.ac.uk/interpro/search/sequence-search)^34^. Phylogenetic analysis of Ppal15kDa and homologous sequences was performed using the BLOSUM series matrix of ClustalW alignments and the neighbor-joining method^54^ with 1000 bootstrap replicates by the Molecular Evolutionary Genetics Analysis (MEGA) version 6.0 software^55^.

### Isolation and cloning of *Ppal15kDa*

Total RNA was isolated using the RNeasy Plant Mini Kit (Qiagen, CA, USA) according to the manufacturer’s protocol. *P. palmivora* mycelium was ground to a fine powder with liquid nitrogen in a mortar with a pestle. The contaminating genomic DNA was removed using an on-column RNase-free DNase I digestion set (Qiagen, CA, USA). First-strand cDNA synthesis was conducted using the Superscript III (Invitrogen, CA, USA).

The protein transcript was amplified with the *oligos Ppal15kDa*-F (5′-GTGCGTAGATAACCAACAGACTG-3′) and *Ppal15kDa*-R (5′-GGTTGGGCTCGTTTCATACTAC-3′) targeting 5′ and 3′ untranslated region (UTR)designed based on the sequence of *PLTG_02159*^13^. The PCR reaction was performed using Emerald Amp GT PCR Master mix (Takara, Otsu, Shiga, Japan). The PCR product was separated on 2% (w/v) agarose gel and purified using the Gel/PCR DNA Fragments Extraction Kit (Geneaid, New Taipei City,Taiwan), and ligated into pGEM-T vector (Promega, Madison, WI, USA). The recombinant plasmids were isolated using the AccuPrep plasmid DNA extraction kit (Bioneer, Alameda, CA, USA) and subjected to Sanger sequencing by Macrogen DNA sequencing service (Seoul, South Korea).

### Transient expression of *Ppal15kDa* in *N. benthamiana* via agroinfiltration

The pJL-TRBO-*Ppal15kDaA* and pJL-TRBO-*Ppal15kDaB* plasmids were constructed by cloning DNA fragments of the open reading frames of *Ppal15kDaA* and *Ppal15kDaB* fused with the hexahistidine (His)-tag encoding sequence at the C-terminus into the pJL-TRBO^35^. The primers, *Ppal15kDa*-FPacI

(5′-GCGttaattaaATGCGTATGMTTCAGGTCGTGTTC-3′) and *Ppal15kDa*-RAvrII

(5′GCGcctaggTCA*gtggtgatggtgatggtg*CTCTTGTCGAAGAAGACGCGATG -3′) were used to amplify the DNA fragments. The introduced PacI and AvrII restriction sites are underlined. The italic letters represent the His-tag sequence. The PCR amplification was performed using Phusion High-Fidelity DNA polymerase (New England BioLabs, MA, USA) by preheating at 98 °C for 30 s, followed by 35 cycles of denaturing at 98 °C for 15 s, annealing at 58 °C for 15 s and extension at 72 °C for 30 s, and a final extension step at 72 °C for 10 min. The amplified fragments were digested with PacI and AvrII restriction enzymes and ligated into the pJL-TRBO vector^35^. The ligation products were transformed into *E. coli* strain DH5α (Invitrogen, CA, USA). The pJL-TRBO-*Ppal15kDaA* and pJL-TRBO-*Ppal15kDaB* plasmids containing the correct *Ppal15kDaA* and *Ppal15kDaB* nucleotide sequences were used to transform *A. tumefaciens* stain GV3101 by electroporation. Transient protein expression in *N. benthamiana* was performed using *A. tumefaciens* GV3101 carrying the above plasmids or pJL-TRBO-G expressing GFP^35^ (as a control) as described previously by Khunjan *et al*.^56^.

### Detection of Ppal15kDa transiently expressed in *N. benthamiana* by Western blot

The agrobacterium-infiltrated leaf tissues were collected using a No. 7 cork borer, flash frozen with N_2_ liquid, and then ground to fine powders using a FastPrep-24 homogenizer (MP Biomedicals). The ground samples were extracted with 2x Laemmli buffer, boiled for 5 min and subsequently centrifuged at 13,000 rpm for 5 min. The supernatant was loaded onto 12% SDS-PAGE and transferred onto a polyvinylidenedifluoride (PVDF) membrane (Thermo Scientific). The His-tagged Ppal15kDa proteins were detected using HRP conjugated anti-His monoclonal antibody His-probe (H-3) (sc-8036 HRP, Santa Cruz Biotechnology, INC) and 1-Step Ultra TMB-Blotting Solution (Thermo Scientific).

### Infection of *P. palmivora* on *N. benthamiana* leaves expressing *Ppal15kDa*

Forty-eight hours after agroinfiltration, two 10-μL drops of zoospore suspensions (1 × 10^4^/ml) were inoculated on each *N. benthamiana* leaf with one drop on the half expressing the *Ppla15kDa* and another drop on the other half expressing *GFP* (control). The lesions were photographed at 4 days after inoculation and the lesion areas were measured using Photoshop (Adobe Systems, CA, USA). The mean ± standard errors (SE) of lesion areas calculated from independent leaves (n = 28) was presented.

### Generation of Ppal15kDa mutants using CRISPR/Cas9 gene editing

Generation of Ppal15kDa mutants via CRISPR/Cas9 gene editing was performed essentially as described by Gumtow *et al*.^28^. A 20-nt sequence G151F (5′-GCCAAGCAGAACAACAACAA-3′), which is on the forward coding strand of *Ppal15kDa* and immediately upstream of 5′-CGG-3′, was selected as the sgRNA target sequence. The potential off-targets of G151F were not found in *P. palmivora* genome^31^. Two oligo-nucleotides, *Ppal15kDa*-Crispr_F1 (5′-ctagc***CTTGGC***CTGATGAGTCCGTGAGGACGAAACGAGTAAGCTCGTCGCCAAGCAGAACAACAACAA-3′) and *Ppal15kDa*-Crispr_R1 (5′-aaacTTGTTGTTGTTCTGCTTGGCGACGAGCTTACTCGTTTCGTCCTCACGGACTCATCAG***GCCAAG***g-3′, which consist of the 20 nt sgRNA target sequence (shaded) jointed together with the HH-ribozyme sequence (underlined, with the reverse complement of the first six nucleotides of the 20-nt target sequence shown in bold italic) and necessary nucleotides for cloning (in lower case), were annealed and cloned to NheI and BsaI sites of pCB301TOR-CRISPR^28^. The resulted plasmid was named pCB301TOR-CRISPR-*Ppal15kDa. A. tumefaciens* strain EHA105 containing pCB301TOR-CRISPR-*Ppal15kDa* was used to transform *P*. *palmivora* papaya isolate P1 via Agrobacterium-mediated transformation following the method described previously^45^. Single zoospore transformants were isolated from the initial G418-resistant transformants as described by Ho and Ko^57^. Briefly, zoospore suspensions (1 µl) at a concentration of 1500 zoospores/ml were dropped onto Plich agar. The agar pieces with a single zoospore were transferred onto new Plich agar for growth. The growing mycelium was transferred and grown on 10% unclarified V8 agar containing 15 μg/ml G418 under 12h-light and 12h-dark cycle at room temperature for 5 to 7 days.

To detect the *Ppal15kDa* mutations in single zoospore-derived transformants, genomic DNA was extracted using DNeasy PowerLyzer Microbial Kit (Qiagen, Germany) according to the manufacturer’s protocol. The *Ppal15kDa* from both WT and transformants was amplified with primers *Ppal15kDa*-FPacI (5′-GCGttaattaaATGCGTATGMTTCAGGTCGTGTTC-3′) and *Ppal15kDa*-RKpnl (5′- GCGggtaccTCACTCTTGTCGAAGAAGACGC-3′) using Phusion High-Fidelity DNA polymerase (New England Biolabs, Ipswich, MA, USA). The PCR products were purified using ExoSAP-IT PCR Product Cleanup reagents (Applied Biosystems, Thermo Fisher scientific, US) and sequenced using the same primers. *Ppal15kDa* was also amplified using *Ppal15kDa*-FPacI.

(5′-GCGttaattaaATGCGTATGMTTCAGGTCGTGTTC-3′) and *Ppal15kDa*-RAvrII (5′GCGcctaggTCA*gtggtgatggtgatggtg*CTCTTGTCGAAGAAGACGCGATG -3′), and cloned into the pJL-TRBO^35^ through PacI and AvrII sites as described above. The recombinant plasmids were extracted using QIAprep Spin Miniprep Kit (Qiagen, Germany) and subsequently used for sequencing.

### Virulence assays of *Ppal15kDa* mutants on *N. benthamiana* leaves and papaya fruits

6-week-old *N*. *benthamiana* plants and green mature Sunrise papaya fruits were inoculated with 15 µl droplets of *P. palmivora* zoospore suspensions (1 × 10^5^/ml). To accurately compare the virulence, WT and mutants were inoculated on *N. benthamiana* leaves side-by-side. On each *N*. *benthamiana* leaf (separated by the midvein), one drop of WT zoospores was inoculated on one half and the same amount of zoospores from a mutant on another half. For inoculation on papaya fruits, three drops of WT zoospores were inoculated on one half of the fruit with a mutant on the other half. The inoculated plants/fruits were kept in clear plastic trays with lids on to maintain high humidity for 1 day. Photos were taken, and the lesion areas and lesion diameters were measured from *N*. *benthamiana* leaves and papaya fruits, respectively, at 4 days post inoculation. The results were calculated from twenty *N. benthamiana* leaves (n = 20) and thirteen papaya fruit (n = 13).

### Sporangium size measurement

Sporangia of 7-day-old WT and mutant *P. palmivora* strains growing on 10% unclarified V8 agar were collected with the 1 ml pipette tips, smeared into 15 µl drops of sterile distilled water on glass slides and covered with cover slips. The sporangia were photographed under a light microscope. The sporangium length was measured using INFINITY ANALYZE software. The results were calculated from independent sporangia (n = 120).

### Zoospore germination and germ tube growth assay

For zoospore germination assay, 1-cm diameter circles were drawn on the back of the petri dish containing Plich agar. A 15 µl drop of zoospore suspension (5 × 10^3^ zoospore/ml) of WT and mutants was dropped on Plich agar medium within each circle. Germinating zoospores were counted and photographed twice under light microscope after growing on Plich agar for 4 hours and 24 hours, respectively. The experiment was repeated three times.

### Appressorium induction

Zoospores from WT and mutants were harvested from 10-day-old 10% unclarified V8 agar plates and resuspended to 5 × 10^4^ zoospores/ml in H_2_O. 30-ul droplets of zoospore suspensions were placed on a plastic cover slip and coved with another one and incubated on Plich agar in petri dish at room temperature for 4 hours. Appressorium formation of WT and mutants was observed and photographed under a light microscope. More than 200 zoospores were counted. The experiment was performed twice.

### Gene expression analyses of *Ppal15kDa*

For vegetative hyphae, agar plugs of 7-day-old *P. palmivora* culture were grown in liquid Plich medium^58^ at room temperature in the dark for 7 days. The vegetative hyphae were collected by vacuum filtration^59^. For sporulating hyphae, agar plugs of 7-day-old *P. palmivora* culture were grown in 10% V8 broth for 3 days in the dark and subsequently transferred to 12 h light/12 h dark condition for 4 days^59^. Zoospore suspensions were prepared as described above and zoospores were collected by centrifugation at 13,000 rpm for 1 min. Cysts were prepared by vigorously vortexing the tube containing zoospore suspension on a mixer for 30 seconds^60^. Germinating cysts were prepared by incubating zoospores on water-treated cellophane membrane and collected when about 80% of cysts germinated^60^. For preparation of appressorium-forming cysts, appressorium induction was performed as described above. After induction for 4 hours, appressorium-forming cysts were collected by centrifugation at 13,000 rpm for 1 min. The vegetative hyphae, sporulating hyphae, zoospores, cysts, germinating cysts, and appressorium-forming cysts were crushed to fine powder in liquid nitrogen. The powders were used for RNA isolation using RNeasy Plant Mini Kit (Qiagen, CA, USA). Contaminating genomic DNA was removed with DNA-free kit (Ambion). One μg of total RNA was used to synthesize first-strand cDNAs using SuperScript II reverse transcriptase (Invitrogen, CA, USA). qPCR was performed using SsoAdvanced Universal SYBR Green Supermix (Bio-rad) as previously described^28^. The oligos *Ppal15kDaqPCR*-F (5′-TCAGGTCGTGTTCATGCTTC-3′) and *Ppal15kDaqPCR*-R (5′-TCTGCTTGGCATCTTCTGTG-3′) were used for specific amplification of *Ppal15kDa* gene. Amplification of *P. palmivora β-tubulin* gene using primers described by Gumtow *et al*.^28^ was used as an internal control to normalize the expression of *Ppal15kDa*. The fold change of *Ppal15kDa* expression at various developmental stages relative to *in vitro* grown mycelium was calculated using 2^−ΔΔC^_T_ method^61^. Three technical replicates were performed.

### Statistical analysis

For analyzing the differential expression of Ppal15kDa at various developmental stages and sporangium size measurements of *P. palmivora* WT and mutant strains, the one-way analysis of variance (ANOVA) according to Duncan’s multiple range tests was utilized to determine significance with P ≤ 0.05 and performed by using SPSS Statistics 17.0 software. The lesion area data derived from *P. palmivora* infection on *N. benthamiana* leaves with one half transiently expressing *Ppal15kDaA* or *Ppal15kDaB* compared to another half expressing GFP gene, and the lesion area data obtained from *N. benthamiana* leaves or papaya fruits with one half infected with WT *P. palmivora* compared to another half infected with mutant *P. palmivora* were analyzed by paired t-test (P-value ≤ 0.05) using SPSS Statistics 17.0 software.

## Supplementary information

supplementary information.
