# Supplementary material for: A secreted protein of 15 kDa plays an important role in Phytophthora palmivora development and pathogenicity
Source: Sci Rep. 2020 Feb 11;10:2319. doi: 10.1038/s41598-020-59007-1 (PMC7012922; doi:10.1038/s41598-020-59007-1)

A secreted protein of 15 kDa plays an important role in *Phytophthora palmivora* development and pathogenicity

Sittiporn Pettongkhao[1,2,3], Natasha Navet[2], Sebastian Schornack[4], Miaoying Tian[2,*], Nunta Churngchow[1,*]

[1]Department of Biochemistry, Faculty of Science, Prince of Songkla University, Hat-Yai, Songkhla 90112, Thailand; [2]Department of Plant and Environmental Protection Sciences, University of Hawaii at Manoa, Honolulu, HI 96822, USA; [3]East-West Center, Honolulu, Hawaii, USA; [4]Sainsbury Laboratory Cambridge University (SLCU), Cambridge, UK

[*]Corresponding author

E-mail address: Miaoying Tian *Tel*: 1-808-956-5305 *Email:* mtian@hawaii.edu

Nunta Churngchow *Tel*: + 66 74 288261 *Email:* nunta.c@psu.ac.th

Supplementary figures

Figure S1. Amino acid sequence alignment of Ppal15kDa and its homologs in *Phytophthora* spp.

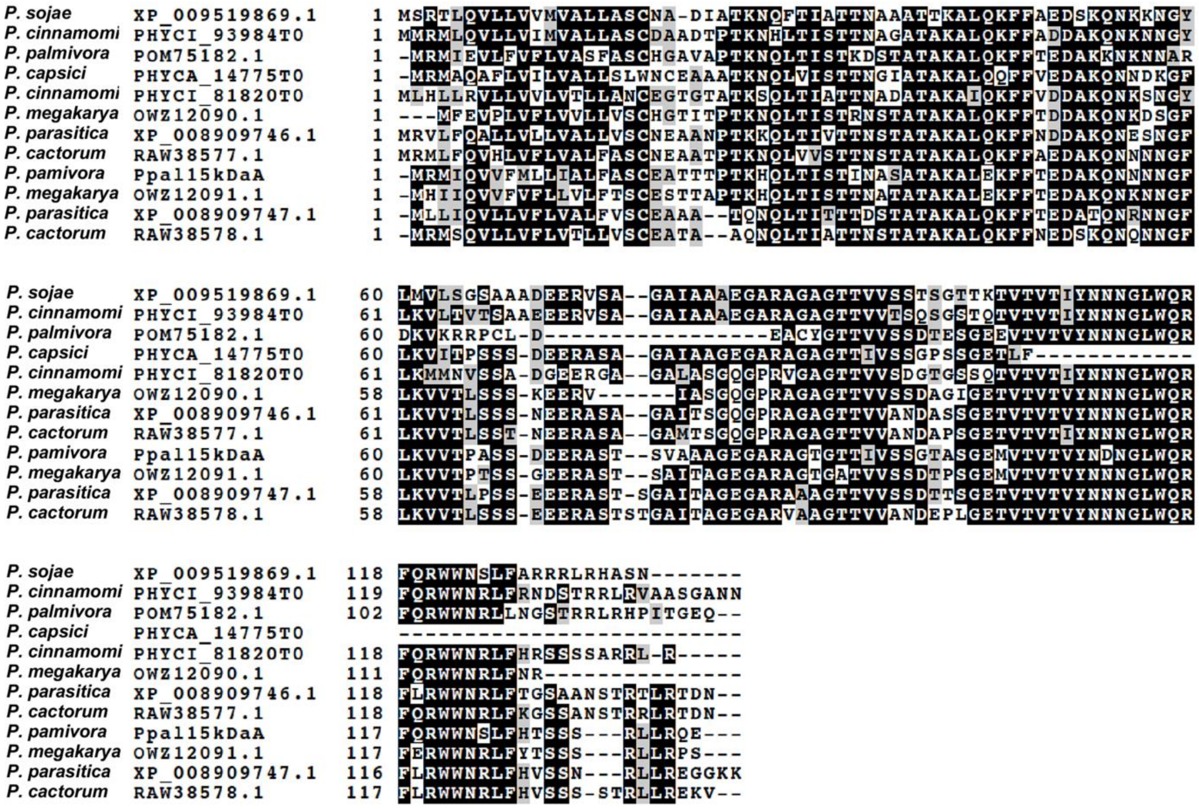

Figure S2. Phylogenetic analyses of Ppal15kDa and its homologs in *Phytophthora* spp. Construction of the phylogenetic tree was performed with the amino acid sequences using the neighbor-joining (NJ) method built in MEGA version 6.0. Bootstrap values were obtained with 1000 replicates and values higher than 50% are shown. The scale bar represents 0.05 amino acid substitutions per site.

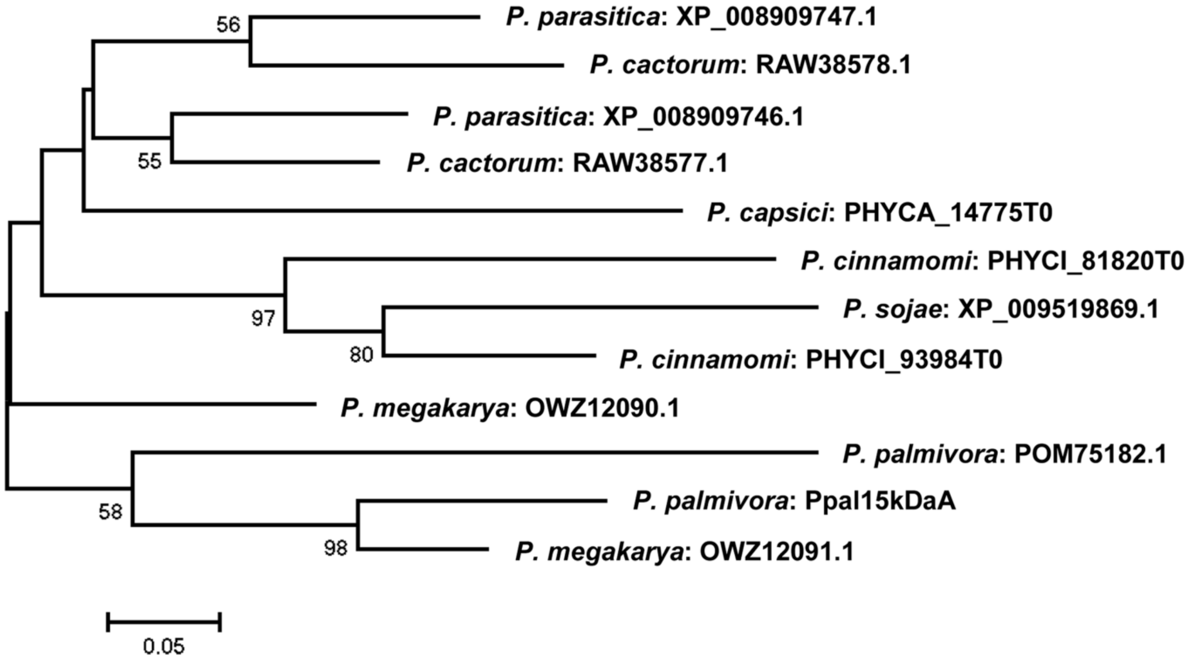

Figure S3. The full-length picture of Western blot analysis of total proteins extracted from infiltrated *N. benthamiana* leaves and subjected to SDS-PAGE followed by Western blot with HRP conjugated anti-His monoclonal antibody. Lane M represents the protein standard and lane GFP represents *N. benthamiana* leaves infiltrated with *A. tumefaciens* GV3101 carrying pJL-TRBO-G. Lane A and B represent *N. benthamiana* leaves infiltrated with *A. tumefaciens* GV3101 carrying pJL-TRBO-*Ppal15kDaA* and pJL-TRBO-*Ppal15kDaB*, respectively. The arrows indicate two forms of Ppal15kDa.

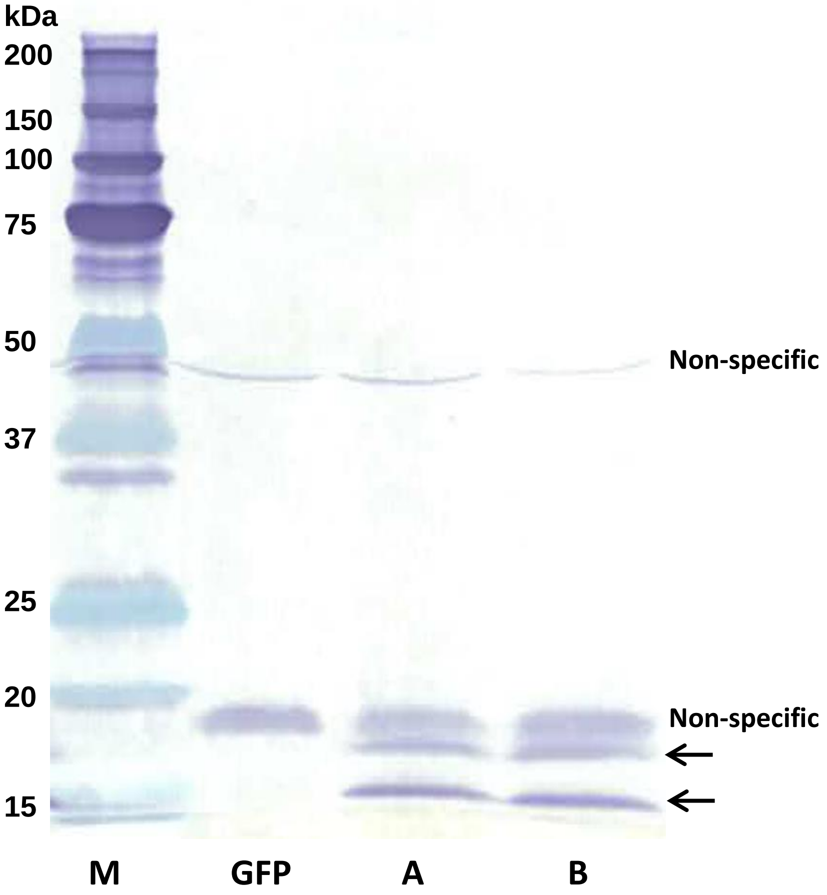

Figure S4. Chromatograms of the partial sequences of *Ppal15kDa* gene in mutants generated using CRISPR/Cas9 gene editing. The mutant lines exhibited mix peaks. The putative Cas9 cleavage site is shown by an arrow.

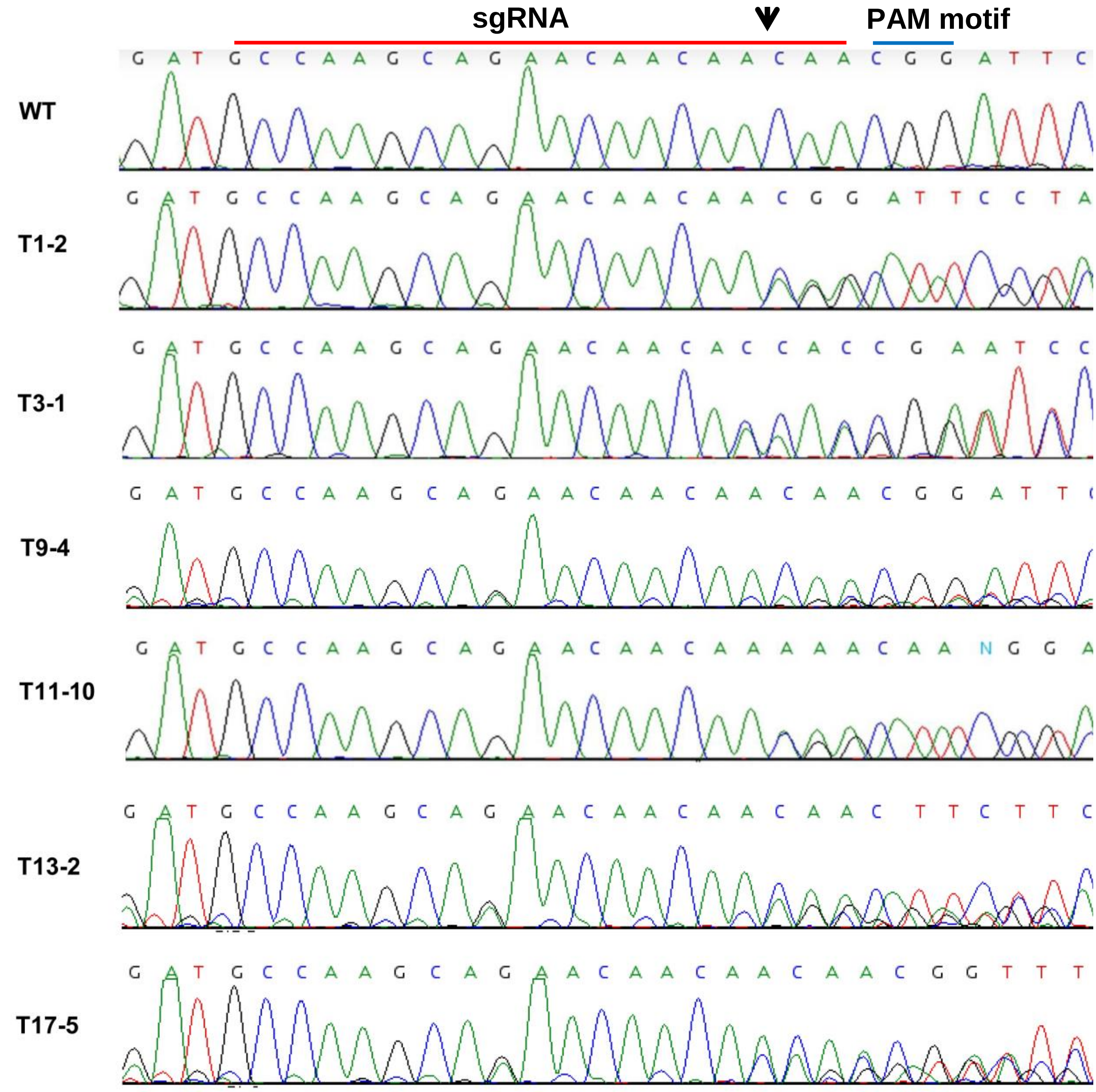

Figure S5. Infection assays of *Nicotiana benthamiana* leaves with *P. palmivora* wild-type (WT) strain and four representative lines that went through transformation but did not have mutations resulted from gene editing.

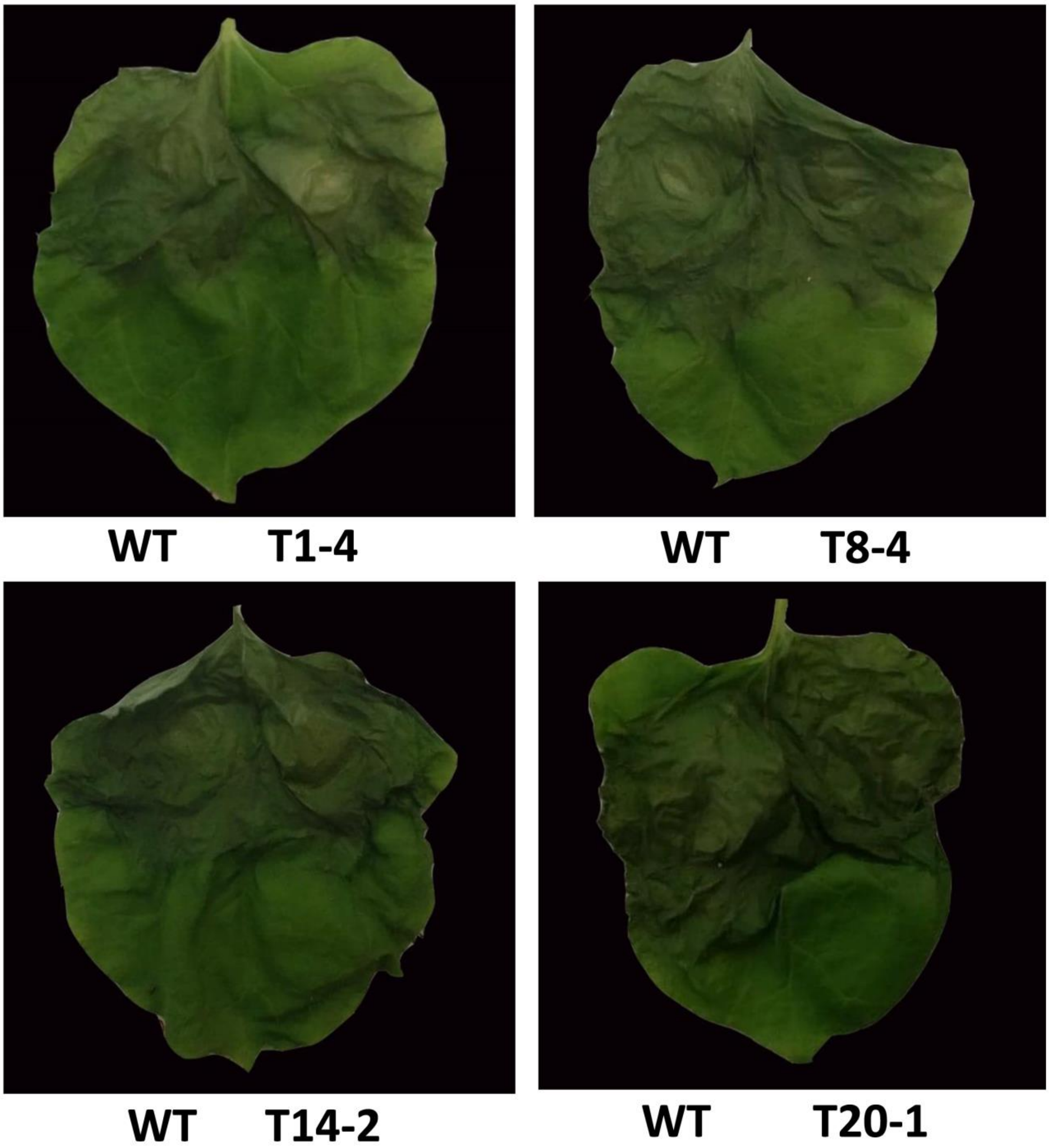

Figure S6. Mycelium growth of WT and mutants. WT and mutants were cultured on 10% unclarified V8 agar. The colony diameters were measured after 5 days.

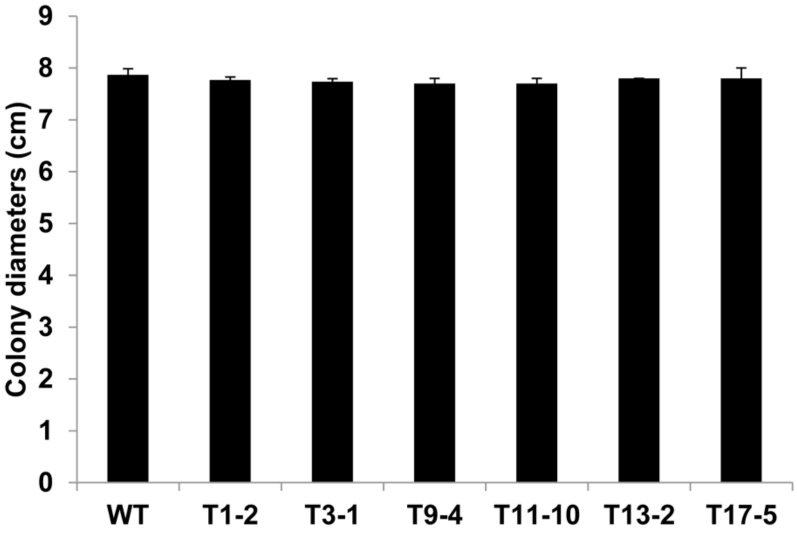

Figure S7. Zoospore germination assay of *P. palmivora* wild-type (WT) strain and *Ppal15kDa* mutants. Zoospores were cultured on Plich agar for 4 hours and photographed under light microscope. Scale bars = 250 µm.

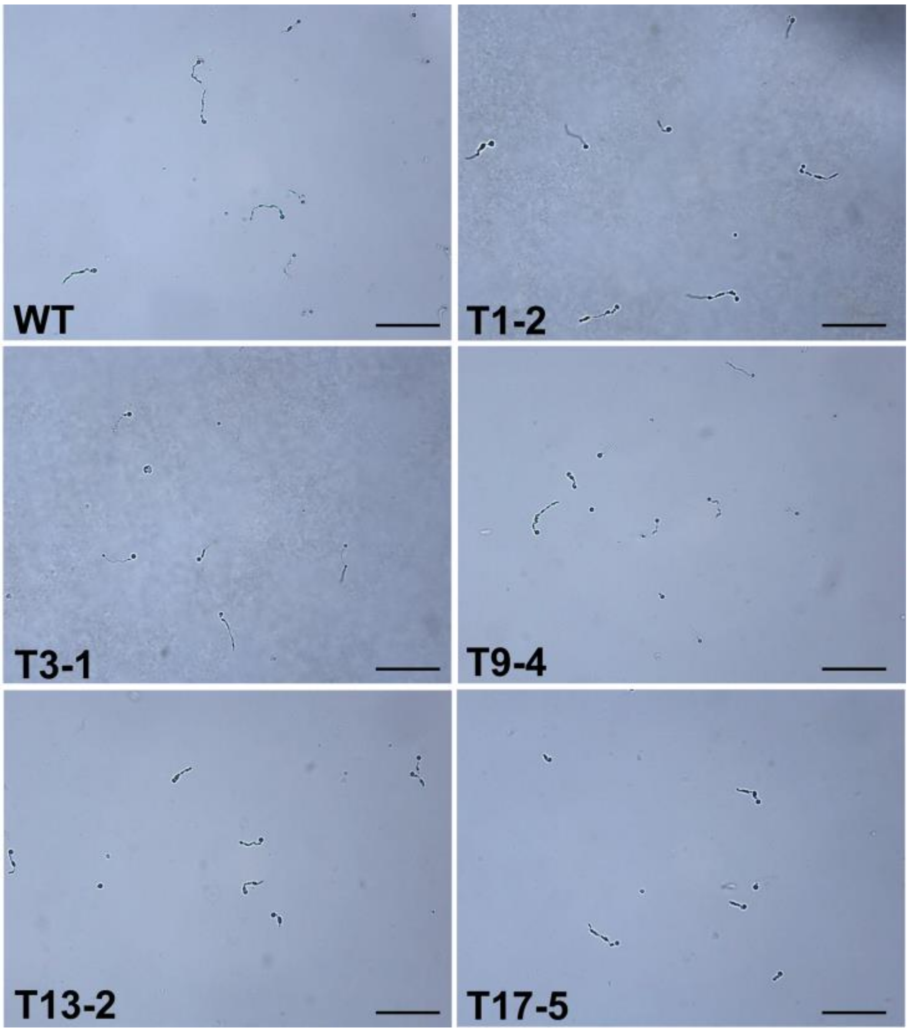

Supplement: Supplementary file 1 — supplementary information. [file 41598_2020_59007_MOESM1_ESM.pdf]
